# Supplementary material for: Personality traits and other factors associated with psychotropic medication non-adherence at two hospitals in Uganda. A cross-sectional study
Source: PLoS One. 2024 Nov 25;19(11):e0302350. doi: 10.1371/journal.pone.0302350 (PMC11588224; doi:10.1371/journal.pone.0302350)
Supplement: Attachment — Submitted filename: Response to reviwers.docx [file pone.0302350.s002.docx]

PONE-D-24-12886

Personality traits and other factors associated with psychotropic medication non-adherence at two hospitals in Uganda. A cross-sectional study.

PLOS ONE

Dear academic editor/PLOS ONE,

Thank you for considering my manuscript and the comments given to improve the work.

I have tried now to follow PLOS ONE's style requirements as it currently stands.

I would like to emphasize that there is no text overlap or duplication from published works or any related previous published works. As you can see using tools for plagiarism (Turnitin for example), the overlap is within the preprint of this manuscript sent to medrixv.org from PLOS ONE during the submission process and is now available on Cold Spring Harbor Laboratory. The preprint has never been published or presented elsewhere and is not under consideration elsewhere.

I would like to address the comments in form of a table which summarizes the comments and how the comments have been addressed.

I look forward for receiving positive feed back.

Kind regards,

Niyokwizera Emmanuel	Principal investigator.

| **Summary of the comments** | **How the comments have been addressed** |
| --- | --- |
| Journal Requirements:  1. Please ensure that your manuscript meets PLOS ONE's style requirements, including those for file naming. | We have adhered to style requirement now. |
| 2.During our internal evaluation of the manuscript, we found significant text overlap between your submission and previous work in the [introduction, conclusion, etc.]. Please revise the manuscript to rephrase the duplicated text, cite your sources, and provide details as to how the current manuscript advances on previous work.  If the overlap is with the authors’ own works: Moreover, upon submission, authors must confirm that the manuscript, or any related manuscript, is not currently under consideration or accepted elsewhere. | The overlap is within the preprint of this manuscript sent to medrixv.org from PLOS ONE during the initial submission process to PLOS ONE and available now on Cold Spring Harbor Laboratory, partner to PLOS ONE.  There is no extract or copy from previous presented or published works.  This manuscript or his preprint has not been published and is not under consideration elsewhere. No related manuscript is under consideration or has been |
| 3. We noted in your submission details that a portion of your manuscript may have been presented or published elsewhere. [No, except a preprint available on research square ". Please clarify whether this [conference proceeding or publication] was peer-reviewed and formally published. | That related preprint was not presented in any conference, was not peer-reviewed or accepted or published in any journal or elsewhere. |
| 4. Your ethics statement should only appear in the Methods section of your manuscript. If your ethics statement is written in any section besides the Methods, please delete it from any other section. | Noted and it has been deleted from other sections. |
| 5. Please include captions for your Supporting Information files at the end of your manuscript, and update any in-text citations to match accordingly. | It has been included. |
| 6. We are unable to open your Supporting Information file [Emma Data 10th November.sa]. Please kindly revise as necessary and re-upload. | It has been revised to XLSL document and re-uploaded. |
| Additional Editor Comments:  Have you encountered any limitations on the impossibility of administering the questionnaire? in what situations? Please indicate this within the limits by expanding this section. Please add further conclusive starting points, such as the involvement of tutors or trustees to reduce the phenomenon of non-adherence to medication. Rewrite the abstract based on the indications provided by the reviewers. | This was an error. The questionnaire was administered by the investigators and research assistant but the responses were more subjective as they were self-report from the patients.  It has been added in conclusion  It has been done |
| Review Comments to the Author  Reviewer #1: The article addresses a current topic; however, there are suggestions that authors should follow to improve the quality of the manuscript. -The psycho-social aspects of medication non-adherence have been little addressed. Are there any prejudices? What are the main factors that can aggravate or weaken a state of mental lability? Poverty? The fragility of an elderly person? The state of abandonment? An unexpected pregnancy? Please expand this discussion by adding further references. | The psycho-social aspect of medication non-adherence has been improved and further references have been added in accordance with these comments.  However, in our study findings, there was no significant association with non-adherence for the additional psycho-social factors mentioned in the comments. |
| -The conclusions are too limited and one topic that is poorly explored is that of the ethical question. For example, is imparting enhanced psychoeducation always correct or could it raise ethical issues, such as the lack of the principle of self-determination and informed consent? Please argue these aspects by referring to further literature | The conclusion has been revised.  The psycho-education is generally done after informed consent is obtained. When well done, the psycho-education can increase motivation and self-determination to change, including adherence to medications. |
| Reviewer #2: Overall, the study provides useful information that highlights non-adherence to antipsychotic medications in Uganda. The study methods and results generally provide adequate context. The focus on the level of psychotropic medication non-adherence and associated personality traits is a strength of the study. However, the authors should consider the following to strengthen it:    BACKGROUND: The following statement seems problematic: "In Sub-Saharan African, by 2050, the burden due to psychiatric disorders and substances use disorders is estimated to increase globally by 130% and specifically by 139% in Eastern Africa if there is no change in the prevalence and management of these disorders (2)." If Sub-Saharan Africa is the focus, is globally needed?" (page 2)  "Patients with psychiatric disorders are more likely to be non-adherent to treatment than those with medical diseases (5)." (page 3). This statement makes it look like psychiatric disorders are not "medical diseases". Consider adding "other" before just "diseases".  The manuscript cites studies in India and Italy about how some personality traits affect non-adherence but similar studies in Africa were not cited. Did the authors not find a single study on antipsychotic medication non-adherence and personality traits in Africa to cite? If no such study exists, then the authors should consider indicating that as a gap in research for which their study aims to partly address. If there are similar studies in Africa too, the authors should consider citing them. | Noted and corrected. Globally has been removed.  Noted and corrected  To the best of our knowledge, no studies on psychotropic medication non-adherence and personality traits in Africa. There is a gap in research in that area. |
| METHODS The authors should consider indicating whether the survey was pilot-tested, and whether patients completed the questionnaire themselves (self-administered). If not self-administered, the authors should indicate whether those who administered received any training to minimise potential bias. How many people administered the questionnaires? | The study was pilot-tested  The questionnaire was administered by 3 psychiatrist and 1 psychiatric clinical officer. They were trained before and received ongoing training during data collection. |
| RESULTS Repetition of "during the study period" in first sentence. Table 1A: How is "Born Again" considered a religion? Did you mean "Pentecostal churches" or similar Charismatic churches? Define abbreviations such as JRRH  TABLE 1B: Define BAD and HIV  TABLE 2: Consider defining cOR under "Bivariate Analysis" as has been done for aOR | Noted and corrected  It has been changed to Pentecostal churches. |
| DISCUSSION In the first paragraph, the authors wrote: "The high prevalence of medication non-adherence found in our study can also be explained by the false beliefs about mental illness and the use of alternative medicine in Uganda, among others factors." However, the study did not assess use of alternative medicines in Uganda. Authors should consider citing at least a study showing that use of alternative medicine contributes to medication nonadherence otherwise they should consider not blaming alternative medicine for nonadherence.  Authors should consider assessing their in-text citation again. It seems references 19 and 20 in the text should be 20 and 21 in the reference list.  Authors should consider avoiding the terms "non-compliance" (pages 3 and 16) as non-adherence is the preferred term.  Authors claim their study is the first in Uganda and in East Africa on the subject (page 16). This is a bold statement.  As indicated earlier, the authors should consider examining the literature on antipsychotic medication non-adherence and personality traits again to determine similar studies done elsewhere in Africa. The can then discuss their findings in the context of the findings of those studies. If they cannot find any such study in Africa, they should consider adding "the best of our knowledge" to qualify that statement on page 16  The strengths and limitations are written like those of theses. Authors should consider rewriting them without bulleted points. | Noted and corrected  Noted and corrected  Noted and corrected  Noted and corrected  Noted and corrected  Noted and corrected |
| ALL OTHER SECTIONS There are several issues with punctuation (eg, missing full stops; page 3 (after adding reference 8); page 7 after below") and inconsistencies that need to be addressed. For example, "non adherence" (several pages) and "non-adherence" (page 3). | Noted and corrected |
