# Supplementary material for: Personality traits and other factors associated with psychotropic medication non-adherence at two hospitals in Uganda. A cross-sectional study
Source: PLoS One. 2024 Nov 25;19(11):e0302350. doi: 10.1371/journal.pone.0302350 (PMC11588224; doi:10.1371/journal.pone.0302350)
Supplement: Attachment — Submitted filename: Response to reviewers 2.docx [file pone.0302350.s003.docx]

PONE-D-24-12886R1

Personality traits and other factors associated with psychotropic medication non-adherence at two hospitals in Uganda. A cross-sectional study.

PLOS ONE

Dear academic editor,

Thank you for considering my manuscript and previous revisions.

I would like to address the additional comments in form of a table which summarizes the comments and how the comments have been addressed.

I look forward for receiving positive feed back.

Kind regards.

| Comment | How the comment has been addressed |
| --- | --- |
| Journal Requirements:  Please review your reference list to ensure that it is complete and correct. If you have cited papers that have been retracted, please include the rationale for doing so in the manuscript text, or remove these references and replace them with relevant current references. | We have reviewed our reference list. It was complete. However, references 19, 25 and 29 were preprints not yet peer reviewed. We have found now their corresponding updated published articles. We have replaced them by the current references. |
| Reviewer #1: The authors have considered the issues raised during the review. However, the Conclusions section continues to be sparse. Therefore, they are again urged to expand it by referring to additional literature | The conclusion section has been expanded by referring to additional literature. |
| Reviewer #2: The authors have to a great extent addressed the issues I raised. However, this manuscript has serious language issues. I am unable to point all of them here.  Under Authors' contributions, the authors have not used proper tenses. They should consult recently published papers in PLOS to learn how to craft Authors contributions. | The English language issues have been addressed with the help of colleagues and English specialists. All the mistakes have been corrected.  The authors’ contribution section has been revised according to PLOS ONE format. |
