# Supplementary material for: Personality traits and other factors associated with psychotropic medication non-adherence at two hospitals in Uganda. A cross-sectional study
Source: PLoS One. 2024 Nov 25;19(11):e0302350. doi: 10.1371/journal.pone.0302350 (PMC11588224; doi:10.1371/journal.pone.0302350)
Supplement: Attachment — Submitted filename: Response to reviewers 3.docx [file pone.0302350.s004.docx]

PONE-D-24-12886R1

Personality traits and other factors associated with psychotropic medication non-adherence at two hospitals in Uganda. A cross-sectional study.

PLOS ONE

Dear academic editor,

Thank you for considering my manuscript and previous revisions.

I would like to address the additional comments in form of a table which summarizes the comments and how the comments have been addressed.

I look forward for receiving positive feed back.

Kind regards.

| Comment | How the comment has been addressed |
| --- | --- |
| Journal Requirements:  Please review your reference list to ensure that it is complete and correct. If you have cited papers that have been retracted, please include the rationale for doing so in the manuscript text, or remove these references and replace them with relevant current references. Any changes to the reference list should be mentioned in the rebuttal letter that accompanies your revised manuscript. If you need to cite a retracted article, indicate the article’s retracted status in the References list and also include a citation and full reference for the retraction notice. | No changes found in reference list. |
| Reviewer #1: All comments have been addressed | OK. |
| Reviewer #2: The authors have slightly improved the manuscript. However, the language issues have not been fully addressed. I am unable to point all of them here, but the authors should consider the following:  1. "The 2017 report of WHO indicated that globally, 80% of people with mental disorders are living in low- and middle-income countries (LMIC) which include Uganda and, up to 75% of affected persons in many LMIC did not have access to the needed treatment [3]." SUGGESTION "are" after "disorders" should be "were", LMIC should be "LMICs", "and, up to" should be ..., and up to" [comma before "and"  "Psycho-social and demographic factors (age, gender, marital status, occupation, family and social support, etc.), substance abuse, beliefs about mental illness are important factors associated with non-adherence related to the patient [5]." SUGGESTION Consider adding "and" before "beliefs".  "Illness related factors like side effects of medications, beliefs about the illness, treatments complexity and lack of insight are barriers to medication adherence [16]."  SUGGESTION "Illness related factors" should be "Illness-related factors [hyphen between illness and related"   The manuscript would need additional editing for issues such as grammar, punctuation and tenses to make its presentation more intelligible. | The Language issue has been addressed.  Corrected.  Corrected.  Corrected.  Corrected.  The manuscript has been fully edited and all errors in grammar, punctuation, and tenses has been addressed. |
